# Developing essential professional skills: a framework for teaching and learning about feedback

**DOI:** 10.1186/1472-6920-5-11

**Published:** 2005-04-01

**Authors:** Penny Henderson, Anne C Ferguson-Smith, Martin H Johnson

**Affiliations:** 1 Department of Anatomy, University of Cambridge, Downing Street, Cambridge, CB2 3DY, UK

## Abstract

**Background:**

The ability to give and receive feedback effectively is a key skill for doctors, aids learning between all levels of the medical hierarchy, and provides a basis for reflective practice and life-long learning. How best to teach this skill?

**Discussion:**

We suggest that a single "teaching the skill of feedback" session provides superficial and ineffective learning in a medical culture that often uses feedback skills poorly or discourages feedback. Our experience suggests that both the skill and the underlying attitude informing its application must be addressed, and is best done so longitudinally and reiteratively using different forms of feedback delivery. These feedback learning opportunities include written and oral, peer to peer and cross-hierarchy, public and private, thereby addressing different cognitive processes and attitudinal difficulties.

**Summary:**

We conclude by asking whether it is possible to build a consensus approach to a framework for teaching and learning feedback skills?

## Background

Notwithstanding many recent changes to the medical curriculum, medical teaching retains a strong apprentice-based element, in which experienced senior doctors pass on their knowledge and skills to students and juniors. The requirements of the profession demand both extensive acquisition of knowledge and a high level of specialist skill development. Multiple academic and qualification hurdles have to be surmounted and there is a highly structured promotion system. This hierarchy of skills and knowledge has consequences for the profession's ethos. First, juniors inevitably know and can do less than seniors. If ineffective or inadequate negative or positive feedback is given, juniors may either develop "false confidence" or become demoralised and fearful of making mistakes. This fear can easily lead to a culture of criticism or blame and so to defensiveness, closing down the junior's openness to learning. Either outcome can be ultimately dispiriting, especially in a highly stressful profession [1,2]. Second, the hierarchical nature of the profession discourages feedback from junior to senior doctors making for a uni-directional "them and us" educational and professional experience. The consequences for the profession of this difficulty in effectively challenging authority figures can be adverse for both the reputation of the profession and the welfare of the patient [3,4]. Given the importance placed on continuing professional development at all levels of the profession, including specifically the requirement for new doctors to be competent in a range of teaching and learning skills [5], the skills of giving and receiving feedback effectively both across and within the hierarchy assume particular importance.

Learning effectively from feedback requires that it is given in a way that helps the recipient to listen to it, receive it constructively, reflect on it, and consider how to take action as a result. Technical aspects of how to give or receive effective feedback after a teaching session have been discussed extensively (see for example [6,7]) and tools are being developed for assessing competence to inform the content of feedback [8,9]. This paper is not concerned with these aspects, which we accept as essential prerequisites for an effective teaching session. We are concerned with how to develop a work culture into which is built the learning and application of the skills required for giving and receiving both positive and negative feedback, regardless of the position of colleagues in the hierarchy, such that the use of these skills becomes second nature and not "an act of bravery" or something to avoid if at all possible. We base our discussion on 14 years of experiential development of a strategic approach to the learning and assessment of the processes and skills of giving and receiving feedback by pre-clinical medical students at Cambridge University (details of the general nature and structure of the course, or of components of it, have been published previously [10-15]). Our objective here is to illustrate some of the key features we have found to be important, as a stimulus for others to share different or similar experiences, and so perhaps to build a consensus approach to a framework for teaching and learning feedback skills?

## Discussion

### Overall structure of the feedback learning process

Initially, feedback was taught as a "technical skill" in a single session and the students were then expected to apply it – the "immunisation" approach. This approach was ineffective and now feedback is taught both conceptually and practically as a reiterative developmental strand throughout a whole year. Table 1 illustrates those course components during which elements of the skills of feedback giving and receiving are addressed explicitly. Key developmental steps in the learning sequence are described below (with reference to Table section in parenthesis).

**Table 1:** Feedback components of the course*

**Structures**	**Skills**	**Attitudes**	**Awareness**
1. "Group contract" for workshops (week 1)	Negotiation	Self & mutual respect	Uses student's 'hopes and fears' to create group safety & self responsibility
2. Group task involving cooperation in a physical activity (week 2)	Distinguish descriptive and evaluative comments	Respect for value of different roles in a group	Awareness of the differing power of types of words and of one's own value assumptions
3. Skill building pairs to develop and share listening/consultation skills (weeks 2–16)	Direct, honest communication; application of theory about communication skills	Trust Openness	Empathy develops awareness of difference; e.g. experiences of being heard influence listening styles
4. Assertiveness exercises (week 4)	Getting to a mutually acceptable outcome; assertiveness	Mutual and self respect	One's own natural style and the impact of stress on it; how to change it or tailor it to situations
5. Explicit feedback exercise (week 7) (see Additional file 1)	Observation Reflection Assertiveness	Respect Resilience	Willingness to take risks; clarify own professional style; mistakes as opportunities for learning
6. Written self evaluation and feedback to one other student after giving a dissertation based seminar to the Department	Synthesis of content and style	Value of clear oral communication	Awareness of strengths and weaknesses of own written and oral communication skills
7. Feedback structure to review video role plays (weeks 12–13)	Identifying specifics; creativity about alternatives	Curiosity about what works and why	Capacity to receive, acknowledge, value & use appreciation and criticism
8. Feedback to course organisers through: 8a. feedback forms about each seminar/talk; 8b. weekly workshop evaluations; 8c. student representation at course management meeting (throughout)	Verbal and written feedback Distinguish opinion from evidence-based description	Risk taking	Feeling entitled to express both appreciation and suggestions to course team
9. Feedback to external examiner (after course end)	Synthesis of observation, evaluation, theory & experience	Self respect	Sensitivity about giving feedback to a senior
10. Summative assessment in practical exam (giving written feedback to a consultant on their consultation skills)	Synthesis of observation, evaluation, theory & experience	Self respect	Sensitivity about giving feedback to a senior

*The course described is taken by third-year pre-clinical students between the completion of their basic science courses and their entry into clinical school. It has 16 weeks of teaching, plus two vacations of 5 weeks each and a period of 5 weeks for independent study including research on a dissertation plus follow up reading. The course is assessed summatively by four written papers, a practical examination and two oral examinations, one based around the dissertation and one centred on the demonstration of acquired skills, one of which is "giving and receiving feedback". The course seeks to develop the integration of scientific and evidential knowledge with personal and professional skills. The emphasis throughout is on a sound understanding of theory grounded in practical experimentation and focussed through structured reflection. The aim is to foster integrated and enduring personal and professional development.

### Developing useful feedback tools

#### (i) Building respect, responsibility and empathy (1)

The value of effective feedback is exemplified at their first session, when the students build their own guidelines for working as a "professional development group". This process is structured to encourage identification of their hopes for learning on the course and their concerns about what might prevent learning. From these shared hopes and concerns, they negotiate a working list of behaviours to maximise hopes and manage concerns. A crucial element that emerges during this process is a requirement to treat each other with respect regardless of their individual level of experience, knowledge and difference (ethnicity, gender, religion, sexuality etc). Respect is a crucial element underpinning the ability to give and to receive feedback. Because this process is shared and student-driven at the outset, they take responsibility for the culture and outcome of course sessions. It also encourages students from the outset to address their own behaviour and attitudes and those of their colleagues and teachers critically, constructively and empathically within an agreed learning framework, and serves as a basis for the later specific development of feedback skills.

#### (ii) Distinguishing description from evaluation (2)

Whilst students understand the distinction between observation and interpretation when looking at, for example, pathological sections or clinical signs, they have more difficulty doing so when observing human behaviour. The conflation of the two in their every day experiences makes an analytical approach to human behaviour a key learning step to effective feedback. An early exercise, in which groups of students undertake a videoed physical group activity, which is then played back to them, encourages each student to observe identified peers and record what they see descriptively. They then report back in turn and other students, including those observed, challenge non-descriptive "interpretive" statements and offer alternative suggestions. This exercise raises awareness of the differing power of types of words and of one's own value assumptions and in the process builds respect for the value of different types of role in a group activity.

#### (iii) Assertiveness (4)

Students engage in three exercises to explore attitudes and behaviour appropriate for taking a difficult situation to an effective outcome through direct communication. During the exercises, the students are also encouraged to identify their own "natural" attitudes and behaviour in potentially difficult situations and to determine what might assist them to develop and apply different and even more effective ones. The outcome from this exercise is a list of useful strategies to facilitate effective direct communication. These exercises do not address directly the giving of feedback among group members, but do build skills and encourage attitudes that are useful for doing this such as self and mutual respect, and empathy.

### Feedback across a hierarchy

#### (i) Building confidence and skills interactively in writing (8b)

Each week, students write evaluations of their workshop learning experiences (details in [14]). In these, students include suggestions to the facilitators for different or more effective ways of achieving the workshop objectives or to make explicit to the facilitators when a workshop worked well for them and why and how it worked or did not. Facilitators respond to these evaluations to encourage such inclusions and to develop a good evidence-based evaluation. This procedure illustrates the legitimacy and value of giving feedback to "superiors" and also allows teachers to challenge and encourage students who are reluctant to engage in this risky process. This weekly exercise therefore builds the concept of feedback gently and promotes development of the skills to do so progressively. Facilitators will offer comments to the students about ways they can give suggestions in a manner which is not offensive or patronising and how to distinguish opinion from evidence-based description. This is particularly valuable when students have had a difficult or uncomfortable experience and are conveying both their emotional state (upset) and their suggestions for how the exercise could have been done differently.

#### (ii) Feedback to help organisers and teachers with their own skills (8a & 9)

Further opportunities for feeding back "upwards" are provided in the confidential course critique to the external examiner, completed by all students, and used by course organisers to plan changes for the following year. In addition, all seminars and lectures are followed by completion of a short standard feedback form (occupying 1/4 of an A4 sheet) issued after each teaching session. The form includes a 1–5 score on overall value plus a space for qualitative comments. This small format was developed to address 'feedback fatigue' and overuse of paper, and has resulted this year in feedback rates of 72.5% for term one and 68% for term two (number of students = 18). Comments of great value to both organizers and teachers have been received.

### One-to-one peer-to-peer feedback (3 & 6)

In week 2, the students initiate a weekly "homework" session in the form of pair-work to build and practice a new communication micro-skill (see [13] for details). The relevant point is that, in these pairings, students are also asked to give each other feedback on how their skills are developing and what is more and less effective about their listening and responding skills. Thus, built into the course is a weekly opportunity for mutual feedback with a clear and valued purpose. The feedback function is re-emphasised at the debriefings of the pair work in the subsequent workshop [13]. Each student also gives a seminar about their dissertation, and another is nominated to give them written feedback about their presentation, clearly distinguishing content and presentation issues. Each student submits a self-evaluation, which is submitted for summative examination (see later).

### Giving feedback in public

#### (i) The first group feedback exercise (5)

The group exercise in week 7 represents a critical point in the feedback learning strategy, which most students find demanding. Up to this point, students have functioned in a culture in which inter-individual feedback is encouraged as a useful tool to help learning. In this exercise, we "go public" and ask for the giving and receiving of feedback in small groups of 6–8 plus a facilitator. We first run a group problem-solving exercise after which each student gives and receives feedback on their "performance" in the task to and from group peers. Each then comments how the feedback process has affected them (see Additional file 1). Resulting from this process, students draw up a check-list of behaviours that aid the giving and receiving of feedback. They compare their own lists with published examples. Throughout, and as previously, they are encouraged to be "allies for each other" in the process of learning by describing and praising achievement and by pointing out alternative possibilities to peers for their less successful actions or comments, but this exercise is a critical test of any mismatch between intention and skill acquisition. In particular, the distinction between description and evaluation becomes critically important. One student, who had a difficult experience in making this distinction effectively, wrote about it thus:

"I did not have a particularly enjoyable time and felt uncomfortable for part of it. ....... in the small group feedback exercise in which I was the observer. I will be the first to admit that the way in which I gave my feedback, despite my best efforts to spare people's feelings, was not good. At times, I felt unsupported whilst giving feedback. It was clear to everyone that it was not going well and to further matters, I felt that the facilitator, herself, could have been more supportive. I will be the first to acknowledge that this perception could have been distorted by my own insecurities regarding my performance. Still, the fact remains that the exercise affected my mood for the rest of the day. ....... there were many things that came out of the workshop that I found useful and thought-provoking such as the difficulty of giving feedback and ways in which the destructive capacity of negative criticism can be minimised. I have tried to be honest [in my feedback] and hope that I have not caused offence – that was certainly not my intention. I feel it to be more beneficial if I express my thoughts in this way than if I were to write an evaluation along the lines of "this is what we did and this is why it was useful."

This feedback demonstrates how hard it can be for students to engage in this process, and also how effectively they can learn from it as demonstrated by the way this feedback was given to the senior person who facilitated the session.

#### (ii) The second group feedback exercise (7)

A more extended public opportunity occurs towards the end of the course, when a day is spent working with actor patients with whom students take turns to practise brief consultations, each of which is videoed and then replayed. Based broadly but not rigidly on 'Pendleton's rules' for feedback [6,7], each student first assesses their own performance for what they did well, and then the rest of the group add their comments. The same sequential process is repeated to garner ideas for what they could do differently. The process emphasises that criticism, which comes with a specific suggestion for alternative behaviour can, under the right conditions, feel more like a gift than an attack. In this session, students display the steady build of giving/receiving feedback skills that has occurred throughout the year as reflected in their feedback evaluations.

"....... the 'doctor' was given a chance for self-analysis first and then was able to see how realistic they were in their praise and criticism of their interview. In most cases we found that we were overly critical of our own performances and that some skills we thought we were carrying out badly or over-using were in fact deemed good or useful by the others." (*Value of feedback as an independent appraisal of own skills*).

"When I make mistakes I become very aware of the thing I did wrong, so the constructive criticism I received after my consultation was invaluable to me. Since beginning this course I find that I welcome feedback, negative as well as positive, whereas before I was scared of it. Being able to improve after mistakes or listening to other suggestions on your current approach, I think, is a very important skill to have."(*Ability to use criticism constructively*).

"I was happy to see an improvement in my "performance" throughout the day and this was much assisted by having specific feedback. Such feedback allows us to learn from our mistakes as well as from others, and could be seen as mirroring the reflective practice that I hope to achieve in my career. Again the way in which we should give and accept feedback was reinforced in the exercises. We should offer it as suggestions of things that could be done differently as opposed to better, (the latter is purely opinion), and we should be as specific as possible. In fact, I often find that receiving feedback that addresses the process of how to change is particularly effective. We need only accept feedback that we agree with and that we can comfortably change, unless it detracts from the care of our patients. The initial comments I received regarded the speed of my speech, phrasing of some question and fidgeting. Just having an awareness of this allowed me to correct these the second time I had a go." (*Recognition of different types and values of feedback and how to use them rapidly to make and monitor change*).

"I've realised just how useful examples can be, in convincing me of the validity of both complimentary and critical comments, so I make sure to try and use them when commenting on others."(*Clear distinction between observation and opinion, and value thereof*).

### Summative assessment (10)

During the year, teaching sessions are not subject to summative assessment and thus provide a space to take risks and develop skills. However, it is made clear at the course outset that the ability to give and receive feedback will be examined summatively. This is achieved in two ways. First, a practical exam consists of a video of a consultation. The students are required to analyse the overall structure of the consultation and the effectiveness with which communication skills were used within it. They are also asked to write feedback to the Dr to help them to develop their skills. The feedback skills demonstrated are assigned a mark equal in value to the analytic section. In 2004, the average mark was 70.4% (range 66% to 80%), where 70% is first class, suggesting that students are performing at a high level. Second, examiners read each student's written self-evaluation of their own seminar performance to assess how well students can distinguish content and presentational skills. Some of these points may be picked up in the oral examination. A final opportunity to assess feedback skills, should this seem necessary, occurs within the oral examinations, students may be offered feedback on their performance from the examiners and some are asked to give oral feedback to the examiners on their examining skills.

## Concluding comments for debate

This paper suggests key criteria for the effective teaching of feedback, with qualitative reports of learning outcomes. Key features are:

(i) early introduction of elements of the skills required, with one-to-one guidance from the teacher, including clear distinction of descriptive from evaluative comments, building of self respect and responsibility for self, and building of mutual respect;

(ii) reiterated opportunities for private peer practice, with generalised group support at intervals;

(iii) a gradual move to more structured written feedback;

(iv) "public" giving and receiving of feedback in groups. This process creates a more developed skill in the communication of both positive and challenging comments to colleagues and superiors. It develops the student capacity to listen attentively and willingly to suggestions and to use them creatively and positively. It encourages self-awareness, since this state promotes the capacity to take responsibility for how feedback is given or received.

(v) By developing an attitude of self respect allied to the values and skills of assertive communication, students build a sense of entitlement to receive the same from others and the skills to ask for it. They may thus later take the risks inherent in offering feedback to colleagues in the health care profession with care and clarity [16], or even to become a whistleblower should the need be unavoidable [17,18].

These conclusions embody many of the principles identified by educational psychologists as key to adult learning, in which the encouragement of specific social feedback from teachers and peers is used to extend and build the students' own feedback skills [19]. It is our contention that, by being open to feedback and used to receiving suggestions, student learning through their remaining professional life should be richer. Additionally, since effective feedback is essentially an aid to change, it may be a particularly useful therapeutic skill for use with patients who are embarking on behaviour or life style change, such as change to dietary, drug or tobacco use [20]. Likewise, patients or their relatives receiving difficult news that requires them to make adaptive changes to accommodate its consequences in their lives may find the process facilitated by clinical students or doctors who have developed their capacity for effective and supportive feedback.

Have the key features we have found to be important shared by others from their experiences, and can we build a consensus approach to a framework for teaching and learning feedback skills?

## Summary

1. Feedback should be taught both conceptually and practically to address attitudes as well as skills, an approach best achieved through longitudinal and reiterative teaching sessions incorporating formative assessment from teachers and peers.

2. The concept of working in mutually respectful partnership towards a shared beneficial outcome, regardless of relative position in the power hierarchy, informs the attitudes underlying feedback giving and thereby the integrity of the practical process.

3. This concept of investment in a shared outcome also reduces the emotional load on both the giver and receiver of feedback.

4. A thorough understanding of the value of distinguishing description from evaluation/interpretation of observed behaviours helps effective technical application of feedback skills.

5. Assessment of feedback skills summatively should be flagged from the course outset and contribute significantly to final grades.

## Competing interests

The authors declare that they have no competing interests.

## Authors' contributions

All authors contributed equally to this work.

## Pre-publication history

The pre-publication history for this paper can be accessed here:



## Supplementary Material

Additional File 1 "Outline of the group exercise used as a prelude to social feedback – as an example". This file describes briefly the exercise referred to in Table 1 as Exercise 5. It describes a group activity that is then used by students to give structured feedback to each other in a public setting. Click here for file
