# Supplementary material for: Developing essential professional skills: a framework for teaching and learning about feedback
Source: BMC Med Educ. 2005 Apr 1;5:11. doi: 10.1186/1472-6920-5-11 (PMC1087486; doi:10.1186/1472-6920-5-11)
Supplement: Additional File 1 — "Outline of the group exercise used as a prelude to social feedback – as an example". This file describes briefly the exercise referred to in Table 1 as Exercise 5. It describes a group activity that is then used by students to give structured feedback to each other in a public setting. [file 1472-6920-5-11-S1.rtf]

Outline of the group exercise used as a prelude to social feedback – as an example

Group members (5-7) are invited to undertake a problem solving exercise, which lasts a maximum of 15 minutes.  Each group has 1-2 peer observers, briefed to make notes of as much behaviour as they can observe, both verbal and non-verbal, with the aim to offer subsequent feedback. The group is told that this is a competitive task with Mars bars for the winning group. The task is described as follows. The group is to tie a reef knot using a magic rope, such that when touched you stick to it. All the group members must engage in the tying of the knot and all must have at least one hand on the rope when the task is completed. Facilitators monitor that the rules are adhered to. The activity is stopped after about 15 minutes or when one or two groups have completed.

Debriefing occurs in the same groups plus a facilitator, who invites each participant, including peer observers, in turn to describe how they felt during the exercise and are feeling now. This round is used to note and discharge any strong feelings, which may have appeared.  Then participants are invited to start jotting down their own DESCRIPTIVE notes about the behaviour they noticed in themselves and their peers during the exercise – and peers also to notice the behaviour of the observer. After 10 minutes, ask them to put these to one side for later use. Next, invite observer(s) to report descriptively, not interpretively, on how they saw each person behaving and the effect that they observed this had on the group task. After this, ask each person in turn to describe how it felt having their actions described, and whether it felt real to them. In particular, they may wish to distinguish and challenge descriptive and interpretative comments.

There is then a short break of 15 minutes, after which they get back into a group with the facilitator. Each person is asked to complete a card legibly for each member of their group (including the peer observer) giving feedback on their behaviour and role in the activity and the impact it appeared to have on group activity.  They put the name of the person to whom it is addressed at the top (personalise it!) and sign it. They have approx 45 minutes for this. The cards are then distributed to the recipients. Each recipient then in turn reads out the feedback, comments on how accurate it seems to them, and on how it affects them to get it. 

The session ends by reviewing the process as a group, teasing out and identifying good features and less helpful ones of feedback given. They are then asked to draw up guidelines for giving and receiving feedback based on their experience. 
